# Supplementary material for: The 2018 European heatwave led to stem dehydration but not to consistent growth reductions in forests
Source: Nat Commun. 2022 Jan 10;13:28. doi: 10.1038/s41467-021-27579-9 (PMC8748979; doi:10.1038/s41467-021-27579-9)
Supplement: Supplementary file 1 — Supplementary Information [file 41467_2021_27579_MOESM1_ESM.pdf]

Supplementary Tables

Supplementary Table 1 | Metadata on sites included within the network. For each site the mean annual temperature (MAT) and mean annual precipitation (MAP) are provided.

Country	Site name	Latitude (°N)	Longitude (°E)	Elevation (m a.s.l.)	MAT (°C)	MAP (mm)	Species (n trees)	Heatwave days
AT	Absamer Vorberg	47.315	11.511	921	7.3	901	*Fagus sylvatica* (3), *Picea abies* (3), *Pinus sylvestris* (3)	10
	Patscherkofel	47.222	11.468	1725	2.8	1202	*Picea abies* (6)	14
	Praxmar	47.153	11.127	1821	2.3	782	*Picea abies* (4)	0
	Tschirgant xeric	47.231	10.844	720	8.6	672	*Pinus sylvestris* (4)	0
	Tschirgant dry-mesic	47.231	10.848	720	8.6	672	*Larix decidua* (4), *Pinus sylvestris* (4), *Picea abies* (5)	0
BE	Experimental forest Aelmoeseneie	50.975	3.804	14	10.6	771	*Fagus sylvatica* (2)	18
CH	LTAL_N08	46.303	7.741	699	9.5	549	*Larix decidua* (4), *Picea abies* (5)	0
	LTAL_N13, LTAL_N16, LTAL_S16	46.392	7.76	1527	4.6	731	*Larix decidua* (9), *Picea abies* (12)	0
	LTAL_N13W	46.393	7.764	1416	5.3	707	*Larix decidua* (2), *Picea abies* (3)	0
	LTAL_N19, LTAL_S19, LTAL_N22	46.39	7.761	1888	2.5	881	*Larix decidua* (12), *Picea abies* (8)	0
	Avegno 01, Avegno 02	46.213	8.744	409	11.4	1416	*Ailanthus altissima* (4), *Castanea sativa* (2)	27
	Alvaneu	46.683	9.641	1233	6.2	750	*Pinus sylvestris* (3)	0
	Neunkirch	47.684	8.534	471	9.9	890	*Fagus sylvatica* (3)	27
	Saillon Buche, Saillon Eiche	46.171	7.166	1031	7.6	802	*Fagus sylvatica* (4), *Quercus pubescens* (3)	19
	Davos Seehornwald	46.815	9.856	1613	3.9	812	*Picea abies* (9)	0
CZ	CZ_J01, ES1000	50.094	17.249	1215	3.6	931	*Picea abies* (6)	7
	CZ_J08	50.064	18.258	1214	9.3	674	*Picea abies* (4)	13
	CZ_SB3	48.992	13.821	1225	4	1125	*Picea abies* (4)	5
	CZ_SJ8	49.167	13.189	1263	3.7	1406	*Picea abies* (3)	18
	Habruvka, Bilovice, Utechov	49.274	16.683	385	8.7	581	*Picea abies* (3), *Quercus petraea* (5), *Fagus sylvatica* (3)	21
	Rajec_old, Rajec_young	49.444	16.696	639	7.1	738	*Picea abies* (7)	21
	Rovna	49.485	16.615	325	9	517	*Picea abies* (3)	26
	Vidly	50.106	17.267	807	5.6	730	*Picea abies* (3)	6
	Bily Kriz	49.502	18.537	855	5.6	1198	*Picea abies* (8)	5
	Stitna nad Vlari	49.036	17.97	557	7.7	815	*Fagus sylvatica* (8)	8
	Lazy	50.043	12.625	828	5.7	882	*Picea abies* (6)	25
	Benesovice	49.742	12.861	514	7.8	693	*Pinus sylvestris* (6)	13
	Zelivka	49.675	15.23	475	8.1	709	*Picea abies* (6)	14
DE	Eldena	54.078	13.476	10	8.9	565	*Acer pseudoplatanus* (2), *Fagus sylvatica* (7), *Carpinus betulus* (4), *Quercus robur* (4)	0
	Vilm	54.326	13.54	10	8.8	618	*Acer pseudoplatanus* (5), *Fagus sylvatica* (4), *Quercus robur* (5)	0

FR	CHS01	46.171	5.238	277	12	1189	*Quercus petraea* (6)	20
	CHS58	46.97	3.66	269	11.2	1041	*Quercus petraea* (10)	11
	CHS72	47.795	0.38	165	11.1	733	*Quercus petraea* (9)	0
	CHS81	44.04	1.75	325	12.5	833	*Quercus petraea* (5)	5
	FRFon	48.476	2.78	98	11.2	699	*Carpinus betulus* (1), *Quercus petraea* (11)	13
	Cheze	42.917	-0.033	1004	9.3	1162	*Quercus petraea* (10)	0
	Laveyron	43.776	-0.217	132	13.8	969	*Quercus petraea* (9)	0
	Charmes	48.373	6.293	274	10.3	849	*Quercus petraea* (10)	11
	Champenoux	48.721	6.34	232	10.3	760	*Quercus petraea* (5)	11
	Ban	48.104	6.21	427	9.7	1066	*Fagus sylvatica* (5)	6
IE	Dooary Forest	52.949	-7.264	245	8.9	1099	*Picea sitchensis* (4)	26
IT	Matsch_SF1	46.678	10.578	1222	6.2	606	*Larix decidua* (3), *Pinus nigra* (4)	0
	Matsch_SF2	46.694	10.613	1766	3.1	690	*Larix decidua* (4)	0
	Matsch_SF3, Matsch_SF5, Matsch_WgS	46.729	10.668	2049	1.5	690	*Larix decidua* (12), *Pinus cembra* (4)	0
	Matsch_SF4, Matsch_WgN	46.679	10.644	2371	-0.4	937	*Larix decidua* (8), *Pinus cembra* (3)	0
NL	Vijlnerbos	50.766	5.969	191	9.7	895	*Fagus sylvatica* (5)	13
PO	BF_03, BF_05, BF_27, BF_33	52.652	23.746	166	7.4	606	*Pinus sylvestris* (9), *Alnus glutinosa* (3), *Quercus robur* (3), *Picea abies* (2)	7
	BF_09, BF_10, BF_12	52.77	23.741	172	7.3	604	*Betula spp* (3), *Tilia cordata* (4), *Fraxinus excelsior* (4), *Acer platanoides* (3)	7
	BF_15, BF_21, BF_24	52.738	23.851	169	7.4	615	*Quercus robur* (3), *Acer pseudoplatanus* (4), *Ulmus spp.* (3)	7
	BF_17, BF_19, BF_20	52.79	23.874	161	7.4	602	*Betula spp,* (3) *Pinus sylvestris* (3), *Fraxinus excelsior* (3), *Alnus glutinosa* (4)	7
RO	RO_BEL_002_2, RO_BEL_004_1	45.638	24.966	1391	3.8	1004	*Fagus sylvatica* (2), *Abies alba* (1)	6
	RO_FA9_457	45.572	24.605	1502	3.3	746	*Picea abies* (3)	6
SK	SK_SRA_008_1, SK_SRA_010_2	49.188	19.109	859	5.6	872	*Abies alba* (2), *Picea abies* (3), *Fagus sylvatica* (6)	0

Supplementary Table 2 | Comparison of tree water deficit during the 2018 heatwave and annual growth in 2018 relative to control years (2016-2017). Linear-mixed effect model output for the ratio of the minimum and maximum tree water deficit during the 2018 heatwave compared to control years (log_{10}[min. $TWD_{2018:control}$] and log_{10}[max. $TWD_{2018:control}$]) and annual growth in 2018 compared to control years (log_{10}[$GRO_{2018:control}$]). Taxonomic clade was tested as a fixed effect to evaluate differences between broadleaves and conifers, but discarded as it did not affect any of the log response ratios. Species and site were considered crossed random effects. For the fixed effects, parameter estimate, estimated degrees of freedom (df), standard error (SE), the *t*-statistic, and the associated *P*-value of significance are given. For the random effects, variance, standard deviation (SD), and the associated *P*-value (using rand function) of significance are given. Linear mixed models were fit by REML and Satterthwaite's method was used for t-tests.

**Dependent variable**	**Trees**	**Effects**	**Fixed effects**					**Random effects**		
	**(Sites)**		**Estimate**	**df**	**SE**	***t***	***P***	**Variance**	**SD**	***P***
log_{10}(min.		Intercept	0.2473	20.14	0.058	4.258	0.000			
$TWD_{2018:control}$)	175	Site (int.)						0.045	0.211	0.000
	(37)	Species (int.)						0.017	0.132	0.023
		Residual						0.063	0.251	
log_{10}(max.		Intercept	0.2065	19.68	0.042	4.942	0.000			
$TWD_{2018:control}$)	175	Site (int.)						0.024	0.155	0.000
	(37)	Species (int.)						0.009	0.093	0.040
		Residual						0.033	0.182	
log_{10}($GRO_{2018:control}$)		Intercept	-0.0902	21.05	0.060	-1.505	0.147			
	175	Site (int.)						0.048	0.220	0.000
	(37)	Species (int.)						0.014	0.119	0.137
		Residual						0.101	0.317	

Supplementary Table 3 | Statistics on linear-mixed effect model parameters per plant functional type. General statistics on fitted fixed effects for relative extractable water (REW) and vapor pressure deficit (VPD) for the ratio of the daily minimum and maximum tree water deficit (TWD) in 2018 against the control years ($TWD_{2018:control}$). Satterthwaite's method and REML criterion was applied to select model estimates. Superscripts indicate the power of the fitted polynomial coefficients. The random part of the model includes the tree nested within site and species as crossed random (intercept) effects. For each fixed effect, parameter estimate, estimated degrees of freedom (df), standard error (SE), the *t*-statistic, and the associated *P*-value of significance are given. Linear mixed models were fit by REML and Satterthwaite's method was used for t-tests.

Target	Dep. var.	N (Sites)	Trees	Fixed effects	Estimate	df	SE	*t*	*P*
Broadleaf	Daily max.	4563 (15)	81	Intercept	0.003	7.0	0.068	0.047	0.964
	$\log_{10}(TWD_{2018:control})$			REW	-5.230	567.8	0.599	-8.740	0.000
				REW^2	3.231	4038.8	0.331	9.766	0.000
				REW^3	-3.367	3097.1	0.274	-12.274	0.000
				VPD	7.080	4362.2	0.273	25.964	0.000
				VPD^2	-3.343	4480.9	0.211	-15.862	0.000
	Daily min.	4563 (15)	81	Intercept	-0.099	6.9	0.115	-0.860	0.419
	$\log_{10}(TWD_{2018:control})$			REW	-7.329	602.3	0.870	-8.427	0.000
				REW^2	6.294	4118.2	0.482	13.070	0.000
				REW^3	-5.530	3175.8	0.399	-13.857	0.000
				VPD	8.401	4386.3	0.397	21.166	0.000
				VPD^2	-4.253	4480.9	0.307	-13.865	0.000
Conifer	Daily max.	4303 (14)	77	Intercept	-0.116	7.4	0.029	-3.954	0.005
	$\log_{10}(TWD_{2018:control})$			REW	-4.336	3507.4	0.335	-12.936	0.000
				REW^2	1.211	3576.9	0.321	3.769	0.000
				REW^3	-0.508	4225.7	0.248	-2.048	0.041
				VPD	6.325	4245.1	0.239	26.469	0.000
				VPD^2	-2.137	4237.3	0.221	-9.660	0.000
	Daily min.	4303 (14)	77	Intercept	-0.184	2.5	0.060	-3.056	0.070
	$\log_{10}(TWD_{2018:control})$			REW	-3.814	3672.5	0.505	-7.547	0.000
				REW^2	0.860	3750.7	0.484	1.776	0.076
				REW^3	-0.807	4235.1	0.374	-2.156	0.0312
				VPD	8.662	4247.4	0.360	24.050	0.000
				VPD^2	-3.330	4238.4	0.333	-9.985	0.000

Supplementary Table 4 | Statistics on linear-mixed effect model parameters per tree species. General statistics on fitted fixed effects for relative extractable water (REW) and vapor pressure deficit (VPD) are provided for *Fagus sylvatica, Quercus spp. (Q. robur* and *Q. petraea), Picea abies* and *Pinus sylvestris*. Statistics are provided for the ratio of the daily minimum tree water deficit (TWD) in 2018 against the control years ($TWD_{2018:control}$). Superscripts indicate the power of the fitted polynomial coefficients. The random part of the model includes the tree nested within site as a random intercept. For each fixed effect, parameter estimate, estimated degrees of freedom (df), standard error (SE), the *t*-statistic, and the associated *P*-value of significance are given. Linear mixed models were fit by REML and Satterthwaite's method was used for t-tests.

Target	Dep. var.	N (Sites)	Trees	Fixed effects	Estimate	df	SE	*t*	*P*
Fagus sylvatica	Daily min.	2311 (10)	41	Intercept	-0.163	8.6	0.098	-1.664	0.132
	$\log_{10}(TWD_{2018:control})$			REW	-8.640	1827.4	0.845	-10.226	0.000
				REW^2	1.837	2187.0	0.480	3.829	0.000
				REW^3	-3.434	2274.2	0.356	-9.645	0.000
				VPD	6.752	2274.2	0.379	17.834	0.000
				VPD^2	-2.739	2266.2	0.329	-8.315	0.000
Quercus spp.	Daily min.	5067 (13)	90	Intercept	-0.095	12.4	0.068	-1.388	0.190
	$\log_{10}(TWD_{2018:control})$			REW	-22.460	4255.0	0.832	-26.986	0.000
				REW^2	9.799	4965.3	0.415	23.624	0.000
				REW^3	-5.931	4955.2	0.335	-17.696	0.000
				VPD	7.137	4982.8	0.293	24.331	0.000
				VPD^2	-1.706	4975.0	0.242	-7.048	0.000
Picea abies	Daily min.	6185 (16)	111	Intercept	-0.165	8.3	0.039	-4.207	0.003
	$\log_{10}(TWD_{2018:control})$			REW	-4.087	5144.8	0.510	-8.011	0.000
				REW^2	2.846	5621.5	0.422	6.750	0.000
				REW^3	-5.482	6103.4	0.324	-16.917	0.000
				VPD	11.184	6110.1	0.349	32.053	0.000
				VPD^2	-3.174	6086.9	0.306	-10.373	0.000
Pinus sylvestris	Daily min.	1806 (6)	33	Intercept	-0.188	5.2	0.073	-2.570	0.048
	$\log_{10}(TWD_{2018:control})$			REW	-4.884	1456.8	0.561	-8.710	0.000
				REW^2	-1.691	1613.9	0.437	-3.872	0.000
				REW^3	-1.251	1749.2	0.377	-3.315	0.000
				VPD	4.131	1771.7	0.336	12.305	0.000
				VPD^2	-1.960	1769.4	0.302	-6.501	0.000

Supplementary Figures

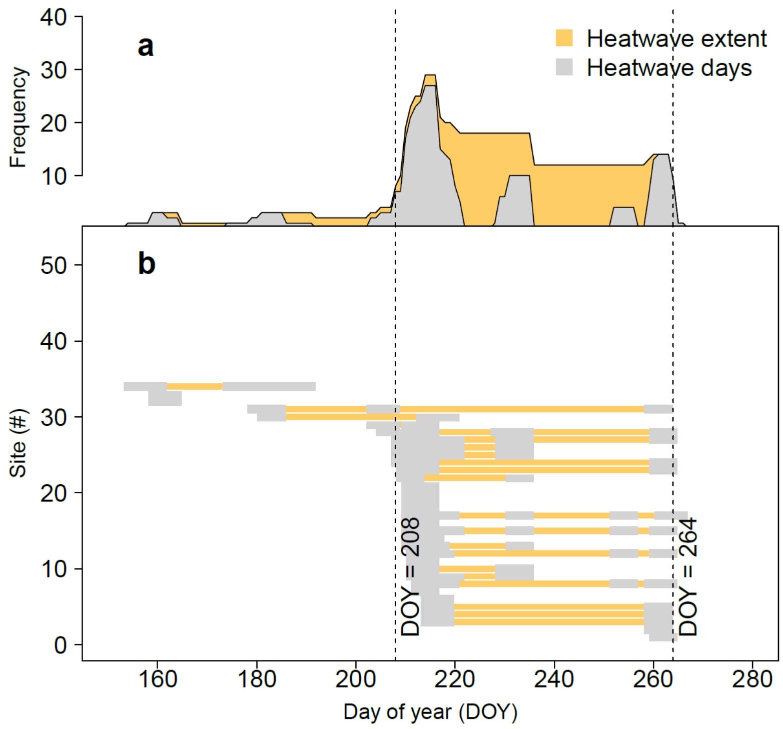

Supplementary Figure 1 | Heatwave extent in 2018 for all sites included within the network. a-b, Frequency distribution and site-specific distribution of the heatwave days and extent. A heatwave is defined as five consecutive days with a maximum daily temperature higher than the 90th percentile of the control period (1951-2000 E-OBS data). The selected overall heatwave extent for analyses is indicated with dotted lines, from day of year (DOY) 208 until 264. This overall extent temporally covers the first and last DOYs when more than five sites overlap in site-specific heatwave days to define the start and the end of the overall heatwave period, respectively.

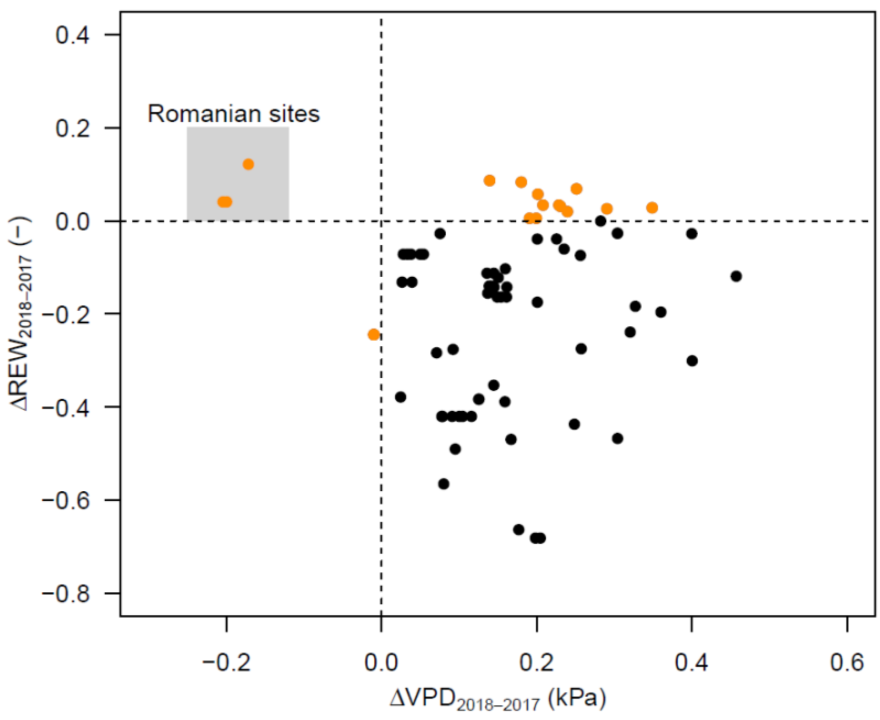

Supplementary Figure 2 | Climatic characterization of sites. Site-specific values of the difference between 2018 and 2017 vapor pressure deficit (VPD) and relative extractable water (REW). For both years, the mean values during the heatwave period (day of year 208 till 265) were determined, and the inter-annual difference (Δ) was calculated. Sites with higher VPD and lower REW in 2018 compared to 2017 are shown in black.

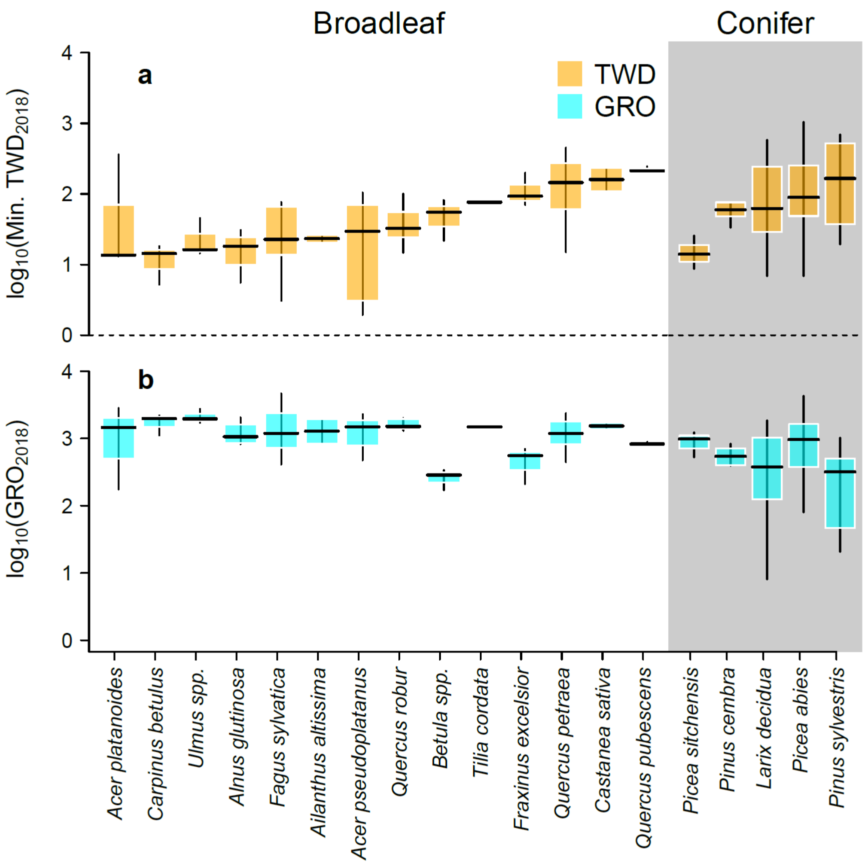

Supplementary Figure 3 | Species-specific response of dendrometer derived parameters in 2018. a-b, Boxplots of log-transformed values of minimum tree water deficit (TWD, µm) during the 2018 heatwave timeframe (Min. TWD_{2018}; a) and annual stem radial growth (GRO, µm) in 2018 (GRO_{2018}; b). Centerlines, box limits and whiskers represent the median, upper and lower quartiles, and extremes excluding outliers (those further than the 1.5 x interquartile range), respectively. n = 175 tree stems over 37 sites.

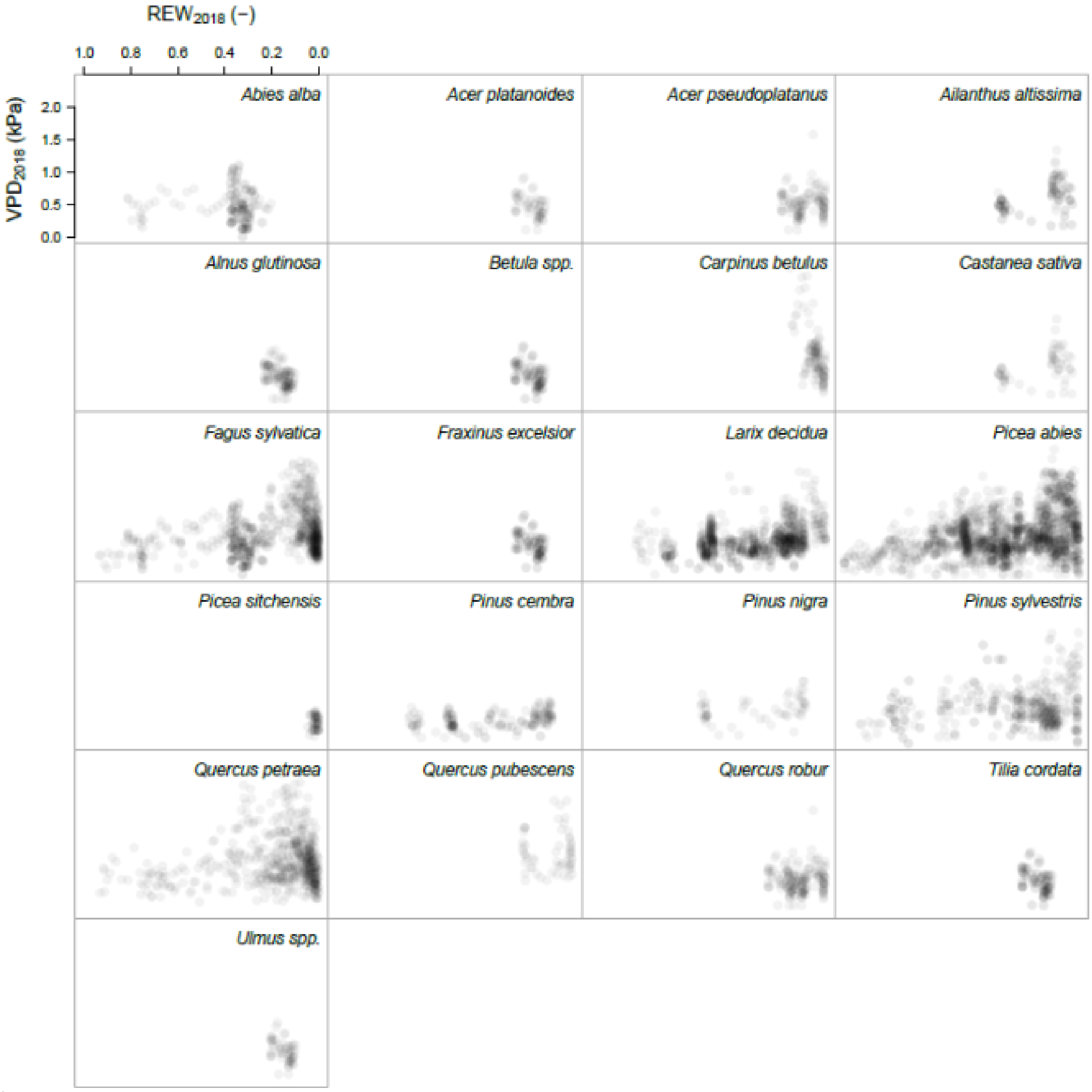

Supplementary Figure 4 | Species-specific hydrometeorological space. Site-specific daily mean vapor pressure deficit (VPD) and relative extractable water (REW) per species during the 2018 European heatwave (from DOY 208 until 264). Darker point clouds indicate a higher occurrence of days with similar VPD and REW conditions.

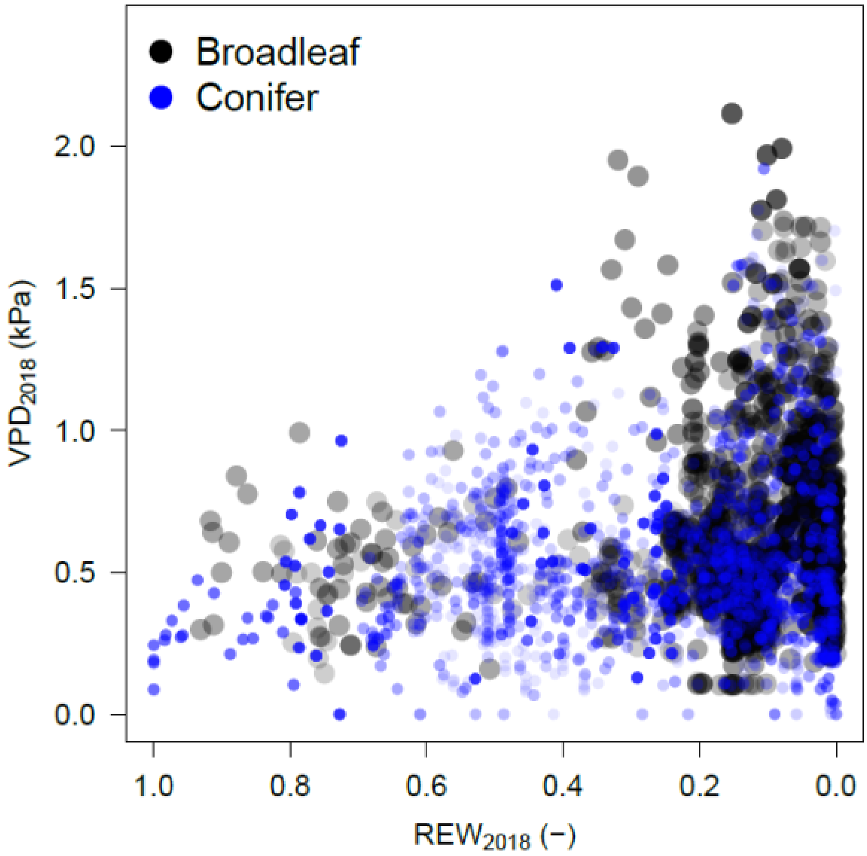

Supplementary Figure 5 | Hydrometeorological space for conifer and broadleaf species. Site-specific daily mean vapor pressure deficit (VPD) and relative extractable water (REW) per taxonomic clade. Darker point clouds indicate a higher occurrence of days with similar VPD and REW conditions.

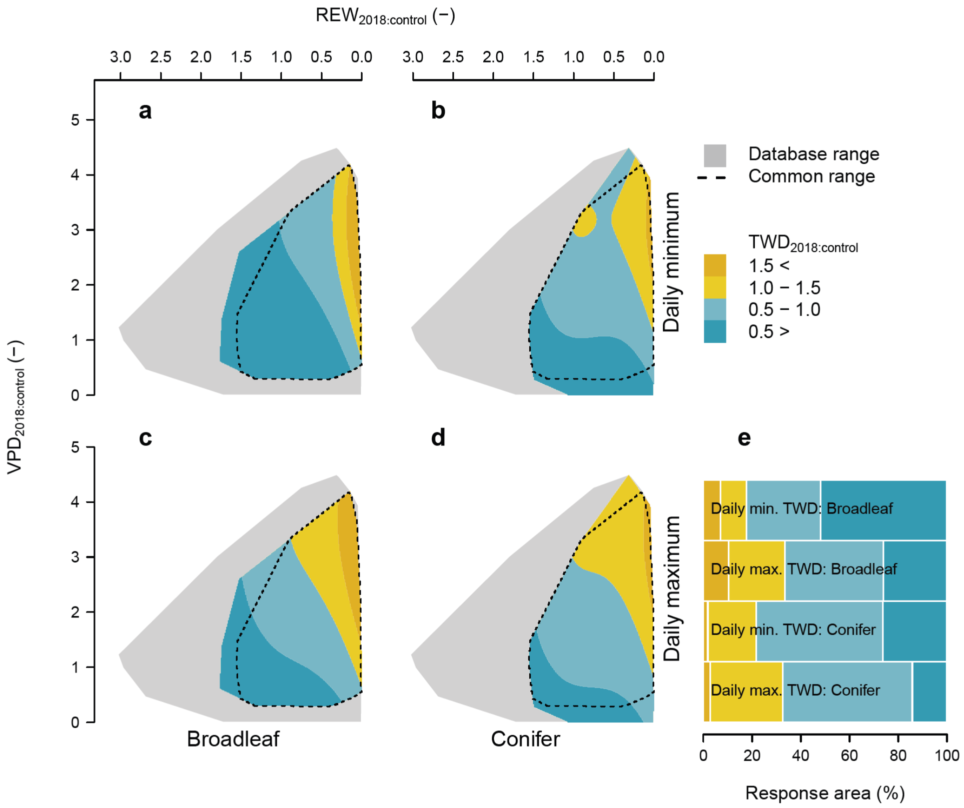

Supplementary Figure 6. Response of tree water deficit ratio ($TWD_{2018:control}$) to the relative change in vapor pressure deficit ($VPD_{2018:control}$) and relative extractable water ($REW_{2018:control}$) during the 2018 heatwave compared to background climate of broadleaf (a, c) and conifer (b, d) species. The hydrometeorological space was determined by dividing the daily 2018 VPD and REW values during the heatwave period (day of year 208 till 264) by the mean VPD and REW conditions during the same heatwave period from 2000 till 2017. **a-d**, Linear-mixed effect model output of the ratio of the daily minimum (a, b) and maximum (c, d) TWD in the 2018 heatwave compared to the 95th percentile of the control period ($TWD_{2018:control}$). $TWD_{2018:control}$ above 1 indicates a larger shrinkage was registered during the 2018 heatwave compared to the control period. From the hydrometeorological space range of the entire database (indicated in grey), models have been adjusted for the common climatic range of broadleaf and conifer species only (indicated with dotted lines). **e**, Bars indicate the percentage of the hydrometeorological space covering different ranges of $TWD_{2018:control}$ values (see legend).

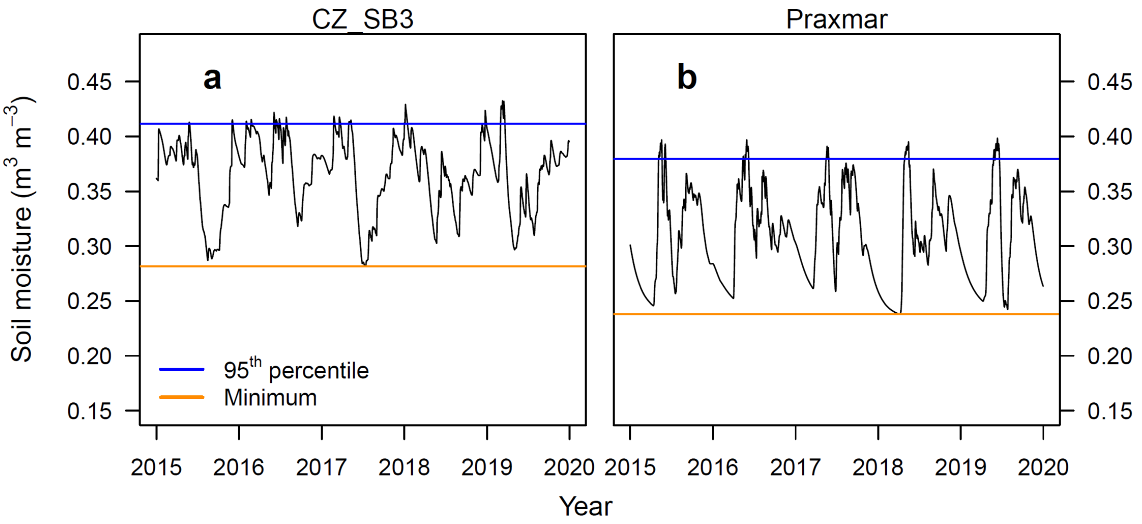

Supplementary Figure 7. Example of soil moisture content time-series for a site in the Czech Republic (a) and Austria (b; see Supplementary Table 1). Relative extractable water (REW) is determined by using the 95th percentile and the site-specific minimum.
