# Supplementary material for: The 2018 European heatwave led to stem dehydration but not to consistent growth reductions in forests
Source: Nat Commun. 2022 Jan 10;13:28. doi: 10.1038/s41467-021-27579-9 (PMC8748979; doi:10.1038/s41467-021-27579-9)
Supplement: Supplementary file 3 — Reporting Summary [file 41467_2021_27579_MOESM3_ESM.pdf]

nature portfolio

Corresponding author(s): Kathy Steppe
Last updated by author(s): Nov 18, 2021

Reporting Summary

Nature Portfolio wishes to improve the reproducibility of the work that we publish. This form provides structure for consistency and transparency in reporting. For further information on Nature Portfolio policies, see our Editorial Policies and the Editorial Policy Checklist.

Statistics

For all statistical analyses, confirm that the following items are present in the figure legend, table legend, main text, or Methods section.

n/a	Confirmed	
☐	☒	The exact sample size (*n*) for each experimental group/condition, given as a discrete number and unit of measurement
☐	☒	A statement on whether measurements were taken from distinct samples or whether the same sample was measured repeatedly
☐	☒	The statistical test(s) used AND whether they are one- or two-sided<br>*Only common tests should be described solely by name; describe more complex techniques in the Methods section.*
☐	☒	A description of all covariates tested
☐	☒	A description of any assumptions or corrections, such as tests of normality and adjustment for multiple comparisons
☐	☒	A full description of the statistical parameters including central tendency (e.g. means) or other basic estimates (e.g. regression coefficient) AND variation (e.g. standard deviation) or associated estimates of uncertainty (e.g. confidence intervals)
☐	☒	For null hypothesis testing, the test statistic (e.g. *F*, *t*, *r*) with confidence intervals, effect sizes, degrees of freedom and *P* value noted<br>*Give P values as exact values whenever suitable.*
☒	☐	For Bayesian analysis, information on the choice of priors and Markov chain Monte Carlo settings
☐	☒	For hierarchical and complex designs, identification of the appropriate level for tests and full reporting of outcomes
☐	☒	Estimates of effect sizes (e.g. Cohen's *d*, Pearson's *r*), indicating how they were calculated

Our web collection on statistics for biologists contains articles on many of the points above.

Software and code

Policy information about availability of computer code

Data collection	No software was used. See data sources below.
Data analysis	- R Core Team. R: A language and environment for statistical computing. R Foundation for Statistical Computing. (2019). R version 3.6.1 (2019-07-05)<br>- Tree-specific dendrometer data were processed using the treenetproc R package (version 0.1.4)<br>- Linear mixed models were adjusted using lme4 (version 1.1-21) and lmertest (version 3.1 -1) R packages

For manuscripts utilizing custom algorithms or software that are central to the research but not yet described in published literature, software must be made available to editors and reviewers. We strongly encourage code deposition in a community repository (e.g. GitHub). See the Nature Portfolio guidelines for submitting code & software for further information.

Data

Policy information about availability of data

All manuscripts must include a data availability statement. This statement should provide the following information, where applicable:
- Accession codes, unique identifiers, or web links for publicly available datasets
- A description of any restrictions on data availability
- For clinical datasets or third party data, please ensure that the statement adheres to our policy

- The dendrometer data and the site metadata datasets analysed during the current used in this study are available in the a public repository (see data availability statement).
- Site-specific meteorological data were compiled using the Global Surface Summary of the Day (GSOD) Weather Data Client (https://joss.theoj.org/papers/10.21105/joss.00177).

- Site-specific soil moisture data were obtained from the ERA-5 land surface model simulations (https://www.ecmwf.int/en/era5-land).
- Long-term climatological conditions were obtained from CHELSA at a 1 km spatial resolution 1 km (https://chelsa-climate.org/).
- Longer daily time-series of the maximum daily air temperature were obtained from E-OBS (spatial resolution = 0.1 °, temporal coverage = 1951-2018) to establish the heatwave timeframe.

Field-specific reporting

Please select the one below that is the best fit for your research. If you are not sure, read the appropriate sections before making your selection.

☐ Life sciences ☐ Behavioural & social sciences ☒ Ecological, evolutionary & environmental sciences

For a reference copy of the document with all sections, see nature.com/documents/nr-reporting-summary-flat.pdf

Ecological, evolutionary & environmental sciences study design

All studies must disclose on these points even when the disclosure is negative.

Study description	To explore the combined effect of drought and heat stress on radial tree growth and stem hydration at a continental scale, we collected a high-resolution, long-term dendrometer dataset from European sites, including the record-breaking 2018 European heatwave (HW2018). The cleaned dendrometer time series were partitioned into growth- and water-related components of stem radius variation (GRO and TWD, respectively). Site-specific meteorological (daily mean atmospheric temperature and vapour pressure deficit) and soil moisture data were compiled in parallel to determine the HW2018 timeframe and perform climate response analyses. Two approaches were applied to analyse data series considering annual and daily temporal scales.
Research sample	We aimed at collecting the largest dendrometer dataset possible meeting requirements for data quality and temporal coverage (see methods for details). As a result, a dendrometer dataset of 377 trees including 21 species from 85 monitoring plots across Europe was compiled.<br>Environmental data at the continental-scale was obtained to explain dendrometer observations. For each site, we extracted time series of daily mean atmospheric temperature (Ta in °C) and vapor pressure deficit (VPD in kPa), obtained from the nearest climate station (search radius = 80 km) using the Global Surface Summary of the Day (GSOD) Weather Data Client. Site-specific soil moisture data were obtained from the ERA-5 land surface model simulations (spatial resolution = 9 km; temporal coverage = 2015-2019; Layer 3: 28 – 100 cm depth). Long-term climatological conditions, including mean annual temperature and mean annual precipitation, were obtained from CHELSA (spatial resolution 1 km). To establish the heatwave timeframe, longer daily time-series of the maximum daily air temperature were obtained from E-OBS (spatial resolution = 0.1 °, temporal coverage = 1951-2018).
Sampling strategy	No sample size was chosen a priori given the nature of this study. The collected dataset is condidered sufficient for our purpose as we covered large gradients of soil and atmospheric drought across European geographic regions, including species with different ecological requirements.
Data collection	Data was collected from different sites using point and band dendrometers located on the bark of tree stems. Data collection was performed by several researchers of the network (see Author contribution statement for details).
Timing and spatial scale	Dendrometer measurements had a temporal resolution of 15-60 minutes. Selected data included three consecutive years (2016 – 2018). Monitoring plots within an Euclidean distance of 9 km, an elevational difference of less than 300 m, and similar soil water conditions were clustered, resulting in a total of 53 sites in Central and Atlantic Europe.
Data exclusions	A quality assessment of the radius time series was performed to assist analyses with specific data-quality requirements, flagging trees with plateauing values during periods longer than 7 days, and temporal gaps larger than 14 days.
Reproducibility	To isolate the temporal variability in GRO and TWD in 2018 relative to control years (2016-2017), we estimated tree-specific ratios of GRO and TWD in 2018 divided by control years. Analyses were reiterated to verify reproducibility of experimental findings.
Randomization	For annual analyses, linear mixed effect models were fitted considering species and site as crossed random (intercept) effects. Correlation between log transformed GRO and TWD ratios was tested likewise.<br>For the climatic (daily) response analysis, linear mixed effect models were fitted to predict min. and max. daily TWD ratios as a function of daily VPD (using a 2nd order polynomial) and REW (using a 3rd order polynomial), with tree nested within site, and species as crossed random (intercept) effects.
Blinding	Blinding was not relevant to this study, as individuals (tree stems) were not subjected to any treatment.

Did the study involve field work? ☒ Yes ☐ No

Field work, collection and transport

Field conditions	See Table S1
Location	See Table S1
Access & import/export	Site access was site-dependent, in all cases performed in a responsible manner to minimize environmental impact. No sampling was performed for this study. Efforts made to access the 53 sites were dependent on the site location.

Disturbance	Depending on the specific bark characteristics, slight debarking was performed to ensure smooth contact between the dendrometer and the tree stem if required.

Reporting for specific materials, systems and methods

We require information from authors about some types of materials, experimental systems and methods used in many studies. Here, indicate whether each material, system or method listed is relevant to your study. If you are not sure if a list item applies to your research, read the appropriate section before selecting a response.

Materials & experimental systems

n/a	Involved in the study
☒	☐ Antibodies
☒	☐ Eukaryotic cell lines
☒	☐ Palaeontology and archaeology
☒	☐ Animals and other organisms
☒	☐ Human research participants
☒	☐ Clinical data
☒	☐ Dual use research of concern

Methods

n/a	Involved in the study
☒	☐ ChIP-seq
☒	☐ Flow cytometry
☒	☐ MRI-based neuroimaging
